# Utilisation of 3D Printing in the Manufacturing of an Anthropomorphic Paediatric Head Phantom for the Optimisation of Scanning Parameters in CT

**DOI:** 10.3390/diagnostics13020328

**Published:** 2023-01-16

**Authors:** Merim Jusufbegović, Adi Pandžić, Mustafa Busuladžić, Lejla M. Čiva, Azra Gazibegović-Busuladžić, Adnan Šehić, Sandra Vegar-Zubović, Rahima Jašić, Adnan Beganović

**Affiliations:** 1 Radiology Clinic, Sarajevo University Clinical Center, 71000 Sarajevo, Bosnia and Herzegovina; 2 Department of Radiological Technologies, Faculty of Health Studies, University of Sarajevo, 71000 Sarajevo, Bosnia and Herzegovina; 3 Department of Mechanical Production Engineering, Faculty of Mechanical Engineering Sarajevo, 71000 Sarajevo, Bosnia and Herzegovina; 4 Faculty of Medicine, University of Sarajevo, 71000 Sarajevo, Bosnia and Herzegovina; 5 Sarajevo Medical School, University Sarajevo School of Science and Technology, 71210 Ilidža, Bosnia and Herzegovina; 6 Faculty of Science, University of Sarajevo, 71000 Sarajevo, Bosnia and Herzegovina; 7 Department of Radiation Protection and Medical Physics, Sarajevo University Clinical Center, 71000 Sarajevo, Bosnia and Herzegovina

**Keywords:** computed tomography, 3D printing, radiation, dose, CTDI, additive manufacturing, imaging

## Abstract

Computed tomography (CT) is a diagnostic imaging process that uses ionising radiation to obtain information about the interior anatomic structure of the human body. Considering that the medical use of ionising radiation implies exposing patients to radiation that may lead to unwanted stochastic effects and that those effects are less probable at lower doses, optimising imaging protocols is of great importance. In this paper, we used an assembled 3D-printed infant head phantom and matched its image quality parameters with those obtained for a commercially available adult head phantom using the imaging protocol dedicated for adult patients. In accordance with the results, an optimised scanning protocol was designed which resulted in dose reductions for paediatric patients while keeping image quality at an adequate level.

## 1. Introduction

Computed tomography (CT) is one of the most used modalities in medical imaging due to its fast performance and high-quality images. The increasing demand for CT examinations has had an extensive impact on the increase in doses of radiation being delivered to patients and is thus a public health concern globally [1,2,3]. Particular concern is given to paediatric patients, whose risk of stochastic effects is increased due to the higher radiation sensitivity of developing organs and tissues, as well as their longer life expectancy. Indeed, imaging parameters should be tailored to keep the radiation dose as low as reasonably achievable, without compromising the image quality necessary to maintain the medical objective. This summarises the concept of optimisation as a key radiation protection principle in medical uses of ionising radiation [4].

One way to approach the optimisation process is to make use of the appropriate imaging phantoms. Ideally, imaging phantoms should mimic the actual size, anatomy, and tissue density of the body part being considered. Due to their likeness to a real patient body, they are referred to as anthropomorphic. Anthropomorphic phantoms replicate anatomical structures of the body and emulate different tissues’ attenuation of X-rays [5]. Many commercially available phantoms are currently in use. However, many researchers have engaged in the production of self-made, personalized, and relatively low-cost anthropomorphic phantoms using different modes of computer-aided design (CAD) and additive manufacturing of three-dimensional (3D) objects, commonly referred to as 3D printing [6,7,8,9,10,11,12].

The accessibility of 3D printers offers flexibility in the production of specific phantoms to be used in biomedical sciences [13,14]. The manufactured phantoms can be used for different purposes, including the optimisation of medical imaging device settings and imaging techniques [15,16].

Information stored in the pixels or voxels of radiological images, such as Hounsfield units (HU), pixel values (PV), or gray values (GV), as well as their size, provide data that could be utilized in the production of phantoms mimicking the human body [17,18,19]. Obtaining this information is the starting point in the analysis of materials used for the printing process [20]. Generally, it is expected for materials to be tissue equivalent, or as close to equivalent as possible. Soft tissues can be mimicked using polymethyl methacrylate (PMMA) and resins, as well as some 3D-printed materials, while gypsum and many other dense materials can be used to simulate cortical bone [21,22,23].

In this study, we described the processes of making an anthropomorphic phantom of an infant patient’s head using adequate materials to simulate various tissue densities. We outlined the process of segmentation of DICOM images, the production of the phantom, and the evaluation of its production quality. Once completed, the phantom was used to optimise CT scanning parameters in the protocol for scanning the heads of infant patients. Therefore, the aim of our study is to analyze and determine whether a novel age-based CT head imaging protocol, calibrated using 3D-printed anthropomorphic phantoms, can be used in clinical practice without compromising image quality.

## 2. Materials and Methods

The optimisation of CT scanning parameters for the infant head protocol was carried out at the Emergency Medicine Clinic of the Sarajevo University Clinical Center (KCUS), utilising a manufactured 3D phantom printed at the Faculty of Mechanical Engineering of Sarajevo University. We used commercially available adult head phantoms and an adult head protocol as reference image quality parameters. In the end, we evaluated the subjective image quality for an unoptimised and optimised scanning protocol.

### 2.1. CT Scanner and Scanning Protocol

In this study, we used a 16-slice Toshiba Astelion (Toshiba, Kawasaki, Japan) CT scanner, which is capable of generating X-rays using different values of tube voltage (*U*), tube current (*I*), rotation time (*t*), pitch (*p*), scan length (*L*), collimation width, beam shaping or bow-tie filter, etc. In general, the selection of these parameters does not change for a specific imaging study, and they are collectively referred to as “scanning protocol”. It is common, however, for *I* to change during the scan, adjusting its value according to the body size and shape. This option is referred to as automatic tube current modulation (ATCM), and it is used in a majority of scanning protocols. Nevertheless, its use for head imaging is not recommended [24].

The most important parameters of the adult patient head protocol at KCUS is summarized in Table 1. The scan length would vary depending on the individual selection by the operator. Other parameters, including *I*, are constant. The recommended “small” bow-tie filter and 320 mm data collection diameter would be used in all patient examinations. Unless individual changes were made, the volume computed tomography air kerma index ($C_{\mathrm{VOL}}$) would be around 65 mGy for all patients, which is close to the diagnostic reference levels (DRLs) adopted in Europe (i.e., Germany and England) [25]. The adult head protocol is an established and accepted CT protocol at KCUS that has produced satisfying image quality [26]. Two series are reconstructed, one suitable for low-contrast soft tissue (i.e., brain) and the other for high-spatial-resolution bone images (i.e., skull). A single head scanning protocol is used for children of different ages (0–16 years), so its image quality and doses are not always adequate. The recommendations, however, suggest the use of different protocols for different age groups [24]. Optimisation of the infant patient protocol (0–1 years of age) carried out within this study represents the first step of protocol optimisation for the child population.

### 2.2. Anthropomorphic Phantom

The anthropomorphic diagnostic and dental head phantom “ATOM Max” (Model 711-HN ATOM Max, CIRS, Norfolk, VA, USA) was used for reference in the image quality evaluation of the adult head scanning protocol. It is composed of materials whose attenuation properties resemble those of normal tissue. Brain tissue (average) consists of carbon (53.60%), oxygen (26.49%), hydrogen (8.16%), nitrogen (1.53%), magnesium (9.98%), and chlorine (0.19%). Cortical bone includes calcium (22.91%), as well as carbon (25.37%), oxygen (35.28%), hydrogen (3.30%), nitrogen (0.91%), magnesium (3.36%), chlorine (0.03%), and phosphorus (8.82%). The density of the two materials are 1.07 g/cm${}^{3}$ and 1.91 g/cm${}^{3}$ for brain and cortical bone tissue, respectively [27]. The images produced should match some of the general characteristics of patient images, mainly, the CT numbers ($N_{\mathrm{CT}}$) expressed in terms of Hounsfield units (HU).

The material used for brain simulation appears to be homogeneous, with no distinction between white and gray matter or cerebrospinal fluid in brain ventricles. Lack of such imaging details is a typical shortcoming of imaging phantoms, especially when knowing that the accurate representation of the gray to white matter ratio could be used for clinical diagnosis [28]. The “ATOM Max” is designed to match an adult patient’s head, with the approximate dimensions of 18 cm×22.3 cm×27 cm and a mass of 6.4 kg [29]. Its use in paediatric radiology is limited. Hence, dedicated paediatric phantoms are being produced and used for the assessment of image quality vs. patient doses [30].

### 2.3. Fabrication of the 3D Model

As stated earlier, phantoms are widely used in the clinical validation and verification of CT imaging systems. However, they are expensive and lack the complexity of the human body’s structure and geometry, which makes the evaluation of imaging methods very limited [31,32]. Additive manufacturing or rapid prototyping, known as 3D printing (3DP), represents an inexpensive solution which not only provides accuracy and accessibility, but also allows for customization [8]. The concept of 3D printing is not new, but recent advances in technology and material selection make a compelling impact in many fields, including medical diagnostics [12,33]. In our study, CT imaging data sets (DICOM) were used for generating patient-specific 3D models of anatomical structures of the head, which was the most demanding stage in the fabrication process. Protected health information was removed from DICOM data sets, so corresponding approval from the local ethical committee was not required. The phantom was printed as a casting mold and filled with gypsum plaster and epoxy resin [34]. Since the DICOM format only represents a two-dimensional image of the 3D layer of the body, a high-quality 3D model was created using the “3D Slicer” software.

**Figure 1:**
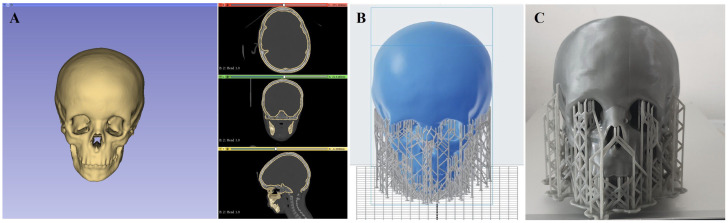
The process of manufacturing an anthropomorphic phantom. (**A**) 3D modeling process using DICOM images in all three planes with bone thresholds; (**B**) After exporting the STL file, it is loaded into PreForms to create supports and prepare for printing; (**C**) the printed anthropomorphic paediatric head phantom with supports.

“3D Slicer” is an open-source software widely used in the medical field, allowing researchers to focus on applications such as communication, visualisation, and data analysis [35]. In order to make a model, we determined the threshold for the bone tissue from the DICOM data set, and then the segmentation process was performed. Figure 1A shows cross-sections of the head image (3 planes) used for threshold determination and segmentation in 3D modeling. After segmentation was completed, volumisation was performed to make the 3D shape of the model. The 3D slicing software itself has an option to visualise the 3D model (Figure 1A). After the model is built, it is exported in Standard Triangle Language (STL) format, a file format native to the stereolithography computer-aided design (CAD) software, and imported into PreForm (FormLabs, Somerville, MA, USA) (Figure 1B), a program for viewing, additional analysis, and 3D printing. The mechanical properties of resins used to make the anthropomorphic phantom were tested before and after printing. Results from examining radiographic characterization of 3D printing materials, which include CT number characteristics of additive manufacturing (AM) materials and data that gave an indication of the uniformity of the material by density on the macro-scale, were detailed previously [20]. The skull was 3D printed with Grey V4 material for high-resolution rapid prototyping. This material is most commonly used for rapid prototyping, product development, and design. Grey V4 is from the “Standard” group of Formlabs resins and is intended for the SLA printing of strong and precise concept models and prototypes with precise details; it has a matte finish and opaque appearance [36].

In addition to the printed casting mold, the resin and hardener (Pan Asel Chemicals Sdn. Bhd Company, Setapak, Federal Territory of Kuala Lumpur, Malaysia) which comprise the epoxy resin were filled in place of the brain and other soft tissue. After the materials achieve enough rigidity, they are ready for further analysis and used in tests that include CT imaging. Plaster is a common building material in the form of a dehydrated powder that, when mixed with water, forms mineral gypsum (${\mathrm{CaSO}}_{4}\;\cdot \;2H_{2}O$). The bone-like mineral composition and density make it a good candidate for mimicking the bone tissue in imaging phantoms production [37]. In this study, we used commercially available modeling plasters (Knauf Gesellschaft m.b.H, Weißenbach bei Liezen, Österreich) whose HU values corresponded to those measured in the skull bones of children.

### 2.4. Assessment of Phantom Quality

The assessment of phantom quality was based on the evaluation of CT numbers in different head regions, as well as true geometrical representation of the skull. Despite its shortcomings and the necessity to put the Hounsfield unit onto a firmer foundation, the following definition is widely used to define CT numbers ($N_{\mathrm{CT}}$) [38]:

(1) $$N_{\mathrm{CT}}=1000\mathrm{HU}\times \frac{\mu_{m}-\mu_{w}}{\mu_{w}},$$

where $\mu_{m}$ and $\mu_{w}$ are the linear attenuation coefficients of the imaged material and water, respectively. From (1), one can derive the relative difference in linear attenuation coefficients (Δμ/μ) of two materials as follows:

(2) $$\frac{\Delta \mu }{\mu }=\frac{N_{{\mathrm{CT}}_{2}}-N_{{\mathrm{CT}}_{1}}}{N_{{\mathrm{CT}}_{1}}+1000\mathrm{HU}}.$$

Initially, we assessed how changes in the scanning parameters *U* (the tube voltage) and *Q* (product of the tube current and the scan time) would affect the image attributes. If we try to image a perfectly uniform object, there is still a variation in the $N_{\mathrm{CT}}$ about some mean value due to the noise. The number of produced and detected X-ray photons depends on both the tube current value (given in mA) and the scan time (given in s). Because of the fact that the tube current and scan time similarly affect noise and patient dose, they are usually considered together as Q=I×t, and it is given in mAs. As a consequence of the energy dependence of μ, we were expecting the absolute value of $N_{\mathrm{CT}}$ to change with beam energy, i.e., in this case, *U*. Based on the above-mentioned analysis, one can conclude, in our case, that the image noise should be affected by both *U* and *Q*.

In our investigation, we measured both average $N_{\mathrm{CT}}$ and σ, which is defined as a pixel’s standard deviation [39,40], inside of a 500 mm^2^ circular ROI in images of adult and paediatric phantoms. To obtain images, we used the scanning protocol for adult heads. The patient dose, which is based on measurements of a 16 cm cylindrical head phantom, was readily available and provided by the manufacturer. Scanning was repeated using different values of *U* and *Q*.

One of the main challenges in this study was to produce a phantom that would represent the anatomy of an infant’s head as close as possible with respect to its dimensions and X-ray attenuation properties, in addition to making it affordable for manufacturing with reasonable but limited resources. Depending on the aim of one’s study, one could make use of different areas of the manufactured phantom for accuracy assessment. Our study aims to optimise patient doses. Hence, we expect the CT numbers of average human tissues (brain, bone, and muscle) to match the doses measured in the phantom, as well as for the phantom’s dimensions to be as close as possible to those of real patients.

**Figure 2:**
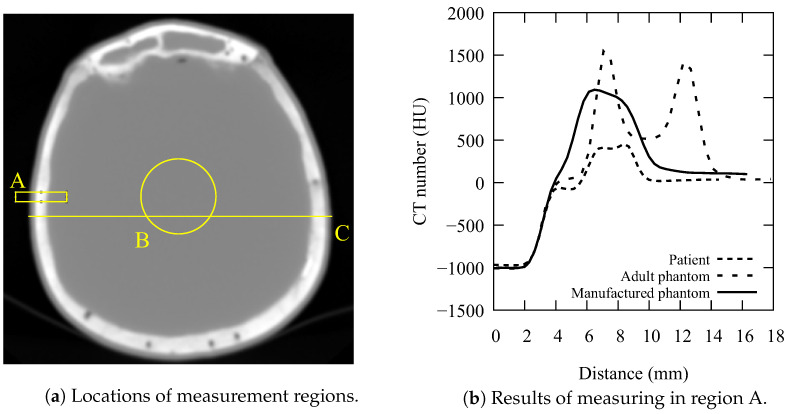
Evaluation of the manufactured phantom accuracy: (**a**) regions of interest used for evaluation of soft tissue and skull (A), brain (B), and lateral size (C); (**b**) profile of 10-pixel average of CT numbers along the *x*-axis in measurement region A. Curves representing CT numbers for patient, adult phantom and manufactured phantom are denoted by dotted, dashed and solid curves, respectively.

Measurement regions used to assess the phantom’s conformity to real patients are shown in Figure 2a. Three different regions, denoted by letters A, B, and C, were chosen. Region A is a 10-pixel-tall rectangle that covers areas in the vicinity of the skull, including the surrounding air, muscle or soft tissue, bone (trabecular and cortical), and brain. The same regions were evaluated on images belonging to a real patient (4-month-old boy), an “ATOM Max” adult head phantom, and the 3D-manufactured paediatric phantom. Remaining regions of interest, denoted by B and C, were chosen to obtain information about soft tissue inside the skull and the lateral size of the skull, respectively. Detailed analyses and explanations will be given in the next section.

### 2.5. Image Quality Model

Developing a new imaging protocol that would be suitable for infant patients required an evaluation of the imaging system’s properties. In this study, we used image noise as an image quality parameter. As we mentioned, in CT images of a perfectly uniform object, there will still unavoidably be a variation in the $N_{\mathrm{CT}}$ about some mean value, due to the fact that interaction of the X-ray photons with matter is a statistical process. This kind of noise is called statistical noise or quantum noise. The standard deviation (σ) of the CT number ($N_{\mathrm{CT}}$) in a uniform region of interest (ROI) and/or square of that standard deviation, i.e., variance ($\sigma^{2}$) are common descriptors of image noise in general. Brooks’ formula [41] states that radiation dose and noise in CT images are connected one to another through the following relation:

(3) $$D\propto \frac{1}{\sigma^{2}}.$$

This relation describes an ideal situation where only quantum noise exists, or where quantum noise is by far dominant over other noise sources such as electronic noise [42] or noise introduced by image reconstruction techniques. To account for the possibility that sources of noise other than quantum noise affect our CT images, we assumed that $\sigma^{2}$ depends on the CT air kerma index C_VOL_ as follows:

(4) $$\sigma^{2}=a+\frac{b}{C_{\mathrm{VOL}}}+\frac{c}{C_{\mathrm{VOL}}^{2}}.$$

The relation (4) was used to model and predict noise for a specific $C_{\mathrm{VOL}}$ after evaluating parameter values using the regression model. Other similar fitting models could also be used [43,44].

### 2.6. Subjective Image Quality Assessment

CT images used in this study were qualitatively evaluated using a 4-point scoring system administered by an experienced paediatric radiologist on the Picture Archiving and Communication System (PACS). Parameters that were evaluated included “noise and image acceptability”, “gray white matter differentiation”, “subarachnoid space acuity”, “visualization of posterior cranial fossa structure”, and “streak artifacts”. The categories in each group were rated 0, 1, 2, and 3, which corresponds to the observation that they are unacceptable, sub-optimal, acceptable/good, and excellent [45,46]. Diagnostic validity is considered acceptable when there is sharpness in the ventricular contours and when the tissue contrast is satisfactory. The differentiation in the gray white matter in the internal capsule, the fourth ventricle, and the corona radiata was evaluated. Sharpness of the subarachnoid space was observed at the ventricular margin, sulci, and cisternal space. Visualisation of the structure of the posterior fossa was assessed by clear visualisation of the sigmoid sinus, vermis, and cerebellum. The classification of streak artifacts is based on the presence of streak artifacts that affect the diagnosis, the presence of streak artifacts that do not affect the diagnosis, and the absence of streak artifacts. Image quality is assessed by observing the most frequent counts in certain categories and the percentage taken for each category [47].

## 3. Results

### 3.1. Accuracy of the Manufactured Phantom

In the first step, we needed to evaluate the quality of the manufactured phantom. Figure 2b shows the measurement results of the Region A. All curves start from −1000 HU (air). The patient curve (dotted line) has a 2 mm-wide plateau at 0 HU, which represents the head’s soft tissue. The second plateau represents the bone tissue, reaching approximately 500 HU, with a small dent indicating the narrow region of the trabecular bone. The curve representing the adult head phantom (dashed line) has distinctive features. While the 2 mm soft tissue plateau is similar to that of a real patient, the area of the skull is represented by two prominent peaks, each representing cortical bone with a CT number of approximately 1500 HU. The trabecular bone is close to 500 HU. In total, this area is 8 mm wide. The manufactured phantom (solid line) is slightly different. The soft tissue area is thinner, while the bone area reaches approximately 1000 HU, a value which is in between those of a real patient and the adult phantom. Just like in the case of a patient, the trabecular bone area is barely visible. The skull is approximately 5 mm thick. With the increase in distance, all curves show constant values that represent brain tissue. It should be noted that skull shape can be different from one patient to another, while skull thickness and composition change rapidly in the early years of childhood [48,49]. Measurements made in Region A provide valuable data regarding the quality of the manufactured phantom. The lack of a 2 mm plateau indicates that the 3D-printed model could be slightly thicker to imitate the soft tissue and skin that surrounds the skull. Indeed, as seen in Figure 1C, the phantom did not include soft facial tissue characteristics, as they were not relevant in the optimisation process of head CT. The bony structures in manufactured phantom, however, resemble those of a real patient, even more so than those of the commercially available adult head phantom. Phantom bones could be further enhanced to exactly match the desired CT number by mixing gypsum plaster with the appropriate amount of other materials (i.e., ground epoxy resin). The two phantom curves in Figure 2b flatten out to a value that closely matches the CT number of brain tissue in paediatric patients. However, Region B provides more information on brain tissue.

The measurement results in Region B were evaluated for different values of *U* and *Q* (Table 2). Both phantoms (adult and paediatric) are solid, with no distinctive features that would represent real brain tissue or pathological conditions. At 120 kV and the highest selected tube loading (Table 2), for the adult phantom, the measured CT number in Region B is (45.3±4.4) HU, while for the manufactured phantom, the CT numbers in this area correspond to (81.6±2.6) HU. The difference is significant (Student’s *t*-test, p<0.001). The differences in CT numbers imply significant differences in the linear attenuation coefficients (μ) of two materials. Nevertheless, the calculations indicate that the relative difference in μ for those two materials, given by Equation (2), is within the range of 3.29–3.51%.

The lateral size of the heads is represented by the maximum width in a selected tomographic projection, as shown in Region C (Figure 2a). The measured widths of the adult and paediatric phantoms are 151.9 mm and 109.6 mm, respectively, while the perimeters are 59.7 mm and 39.9 mm, respectively. The accuracy of the measurements was limited by pixel size, which is 0.468 mm per pixel in the adult image and 0.351 mm per pixel in the paediatric patient image. However, more uncertainties are associated with the changes in skull shape [50].

### 3.2. Protocol Development

Table 2 shows the measurements of CT numbers in the central region of the adult and manufactured paediatric phantom at different values of *U* and *Q* (product of *I* and *t*). The stepwise multiple linear regression analysis (MLRA) was performed in order to investigate which of the parameters, *U*, *Q*, or $C_{\mathrm{VOL}}$, have the greatest influence on image noise, expressed in terms of $\sigma^{2}$. For both the adult and paediatric phantoms, the MLRA showed that only $C_{\mathrm{VOL}}$ was a significant predictor of $\sigma^{2}$, with p=0.002 for the adult phantom and p=0.001 for the paediatric phantom. Two other variables, *U* and *Q*, were excluded as insignificant alone in the MLRA prediction model, with p>0.05.

**Figure 3:**
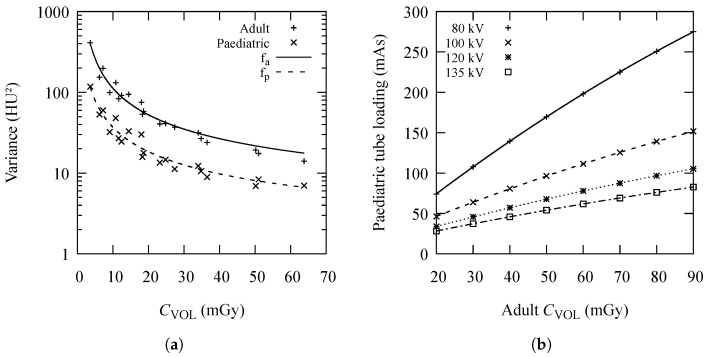
(**a**) Dependence of CT numbers variance ($\sigma^{2}$) on volume computed tomography air kerma index ($C_{\mathrm{VOL}}$) obtained using different tube loadings (mAs) and voltages (kV) on a 16-slice Toshiba Astelion computed tomography unit, measured in the centre of the adult and paediatric head phantom. Coefficients of determination for two fitting curves are $R_{a}^{2}=0.957$ and $R_{p}^{2}=0.965$. Data for adult and paediatric phantom are given by symbols + and ×, respectively, while fitted curves for adult and paediatric phantom data are given by solid and dashed curves, respectively; (**b**) tube loading for paediatric patients at different tube voltages corresponding to adult volume computed tomography air kerma index ($C_{\mathrm{VOL}}$) at 120 kV with the same image noise as the 16-slice Toshiba Astelion computed tomography unit. The four curves account for 80 kV (solid curve with symbol +), 100 kV (dashed with symbol ×), 120 kV (dotted with symbol *), and for 135 kV (dash-dotted with □).

Figure 3a shows how the variance in the CT numbers in the selected ROI depends on $C_{\mathrm{VOL}}$. Images from both the adult and manufactured paediatric phantoms obtained using different values of *U* and *Q* were evaluated. The model (4) was used to represent the regression lines. The fitting parameters *a*, *b*, and *c* for the adult head phantom curve (solid line) are $a_{a}=(3.3\pm 9.9)HU^{2}$, $b_{a}=(890\pm 210)HU^{4}$, and $c_{a}=(2000\pm 760)HU^{6}$, while for the paediatric head phantom (dashed line) they have values of $a_{p}=(1.6\pm 3.3)HU^{2}$, $b_{p}=(320\pm 71)HU^{4}$, and $c_{p}=(330\pm 260)HU^{6}$. The corresponding coefficients of determination are $R_{a}^{2}=0.957$ and $R_{p}^{2}=0.965$, which indicate a very strong dependence of $\sigma^{2}$ on $C_{\mathrm{VOL}}$.

Figure 3b provides a graphic representation of the model. The four curves, one for each value of *U*, indicate the appropriate tube loading for paediatric patients at the desired value of adult $C_{\mathrm{VOL}}$.

In order to obtain the CT air kerma index for paediatric patients ($C_{\mathrm{VOL},p}$) that would produce the same variance as the corresponding $C_{\mathrm{VOL}}$ for adults ($\sigma_{a}^{2}$), the inverse function of (4) needs to be calculated using the following expression:

(5) $$C_{\mathrm{VOL},p}=\frac{b_{p}+\sqrt{b_{p}^{2}-4a_{p}c_{p}+4c_{p}\sigma_{a}^{2}}}{2(\sigma_{a}^{2}-a_{p})},$$

where $a_{p}$, $b_{p}$, and $c_{p}$ are fitting parameters for the paediatric regression curve from Figure 3a.

Based on the results presented in Table 2 and the fact that $C_{\mathrm{VOL}}$ is directly proportional to *Q*, one can now calculate the values of *Q* that would produce the desired $C_{\mathrm{VOL},p}$ at any *U*. A look-up table can be created with conveniently arranged results (Table 3).

For example, if one wants to change the paediatric protocol to lower values of tube voltage (i.e., 100 kV), but maintain the same noise as for adult patients at 120 kV and 50 mGy, the recommended value of *Q* would be approximately 96.4 mAs (Table 3). The new $C_{\mathrm{VOL}}$ will be 16.3 mGy, only one-third of the original dose. Some CT scanners might struggle with the use of high tube currents, *I*. This should be taken into consideration when selecting the appropriate value of *U*.

### 3.3. Patient Doses

**Figure 4:**
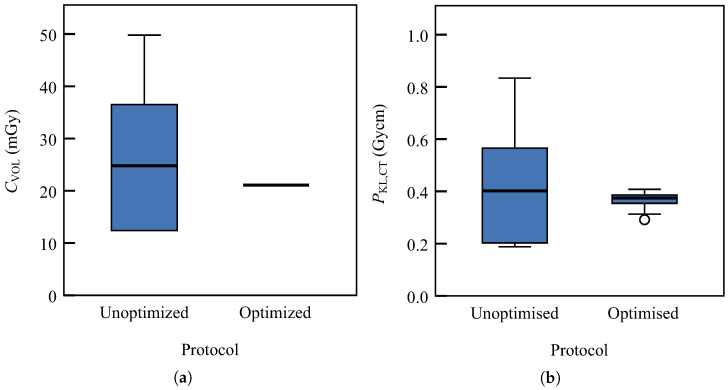
The box plot represents the distribution of dose descriptors for infant patients who underwent head computed tomography examination: (**a**) volume computed tomography air kerma index ($C_{\mathrm{VOL}}$); (**b**) air kerma length product ($P_{\mathrm{KL},\mathrm{CT}}$). In both box plots, the left-hand side stands for unoptimised protocol data and the right-hand side stands for the optimised one. Outliers are represented with circles.

The newly created protocol was tested. Following the baseline of 70 mGy $C_{\mathrm{VOL}}$ at 120 kV for adults, we decided to select U=100 kV, I=170 mA, and t=0.75 s for our paediatric protocol. Table 4 shows average values of observed scanning parameters and dose descriptors, $C_{\mathrm{VOL}}$ or ${\mathrm{CTDI}}_{\mathrm{VOL}}$ and $P_{\mathrm{KL},\mathrm{CT}}$ or DLP. Both $C_{\mathrm{VOL}}$ and $P_{\mathrm{KL},\mathrm{CT}}$ are normally distributed (Shapiro–Wilk test, p>0.2). The two-sample *t*-test indicates significant differences in $C_{\mathrm{VOL}}$ between the unoptimised and optimised protocols (p=0.021). The dose received by the patients under the optimised protocol is significantly smaller. We observe that doses in the unoptimised protocol are widely distributed, with σ=14 mGy. This is clearly visible in Figure 4a. Although a dedicated inquiry could be carried out to investigate the reasons for such a wide distribution, we assume it is caused by the arbitrary selection of scanning parameters. Thus, protocol optimisation was deemed to be necessary. The boxplot of the optimised $C_{\mathrm{VOL}}$ indicates the use of a constant $C_{\mathrm{VOL}}$ in all patient examinations. On the other hand, some deviations exist for $P_{\mathrm{KL},\mathrm{CT}}$, which are caused by varying scan lengths *L* (Figure 4b).

### 3.4. Subjective Analysis

**Figure 5:**
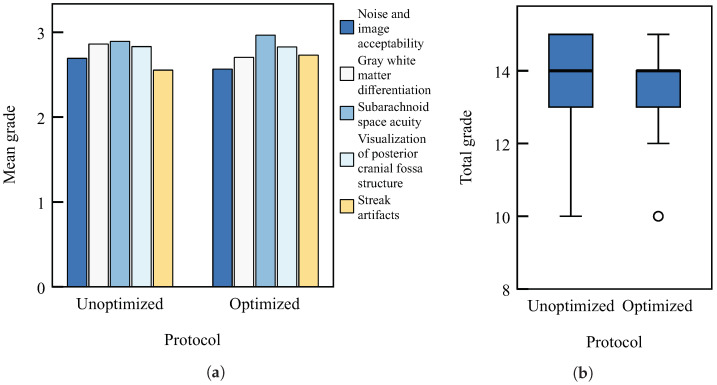
Subjective image quality grades for unoptimised and optimised scanning protocol: (**a**) mean grades of five criteria explained in Section 2.6 of the main text and (**b**) box plot of total grade. No significant differences in quality of patient images using unoptimised and optimised scanning protocol (Mann–Whitney *U* test, p=0.379). In both diagrams, the left-hand side stands for unoptimised protocol data and the right-hand side stands for the optimised one.

The results of the subjective image quality analysis are shown in Figure 5. The mean grades of the five selected image quality criteria are shown in Figure 5a, while the box plot in Figure 5b describes the distribution of the total grades given by the radiologist. The results of the Mann–Whitney *U* test indicated that no significant differences in image quality exist between the unoptimised and optimised protocol (p=0.379), while all mean grades are above 2, which is a grade that represents acceptable/good images.

## 4. Discussion

Exposure to ionising radiation is a health concern in both adults and children. However, the paediatric population is at a greater risk than adults for developing cancer after being exposed to radiation [51,52]. Therefore, it is necessary to adjust paediatric protocols that make use of ionising radiation. One way of adjusting these protocols is taking into account the paediatric patient’s age due to the existence of age-related variations in the size of humans’ anatomical structures. Since 2015, a few studies have been conducted to estimate and optimise the dose received by paediatric patients subjected to CT head examinations. They are dominantly based on the size-specific dose estimate (SSDE) as an approach that is introduced for better-personalized dose estimation [46,53,54,55]. Other investigations include national surveys of radiation exposure in children from CT practice [56,57]. Comparing our optimised $C_{\mathrm{VOL}}$ value of 21 mGy and median or DRL values reported in the above-mentioned studies, one can conclude that our optimised value is below or comparable to other reported median/DRL values. This is also valid for older results published from countries worldwide, as is clearly visible from the upper left diagram of Figure 4 in [57]. In addition, it is important to stress that our optimised value is below the values proposed by the European guidelines on DRLs for Paediatric Imaging published in 2018 [58]. More precisely, in accordance with the guidelines, median $C_{\mathrm{VOL}}$ values of 24 mGy and 28 mGy of DRL distribution were established for the 0–<3 months and 3 months–<1 y age groups, respectively. Both values are greater than the optimised value we obtained for the 0–<1 y age group.

Although the automatic exposure control (AEC) is widely used to reduce radiation dose to patients, its use in head CT protocols has not been clearly defined. Areas where the patient is more rounded and uniform, such as the head, have less adjustment [59,60]. In some cases, the tube current in head CT protocols is modified to only vary along the scan direction of the patient (longitudinal adjustment), while no angular modulation is performed [61]. It is important to realize that AEC techniques will only reduce doses efficiently as much as the specified/desired image quality allows [62]. We also stress that, when using AEC, the radiation exposure depends on the type of AEC software, the parameters input into the software, and the direction of the localizer images [63]. There are facilities where AEC is not used for head/brain CT, which is actually in the majority of the cases in the above-cited literature.

Using different manipulations of the imaging parameters for the CT protocols, the radiation dose delivered to the patient can be significantly reduced. Tube loading (*Q*) and radiation dose ($C_{\mathrm{VOL}}$) have a linear relationship, so reducing *Q* by 50% will reduce the dose by half [64]. In addition to the tube loading, the remaining important parameters are tube voltage, given in kV, and level of iterative reconstruction [39,40]. The last one was not considered in our study.

Image quality parameters, such as image noise, or signal-to-noise ratio (SNR), and contrast-to-noise ratio (CNR), are commonly considered in optimising the CT protocol. However, exposure and reconstruction-related parameters, as well as the tissue to be imaged, should be considered in addition to the image quality parameters to create an optimal protocol for paediatric imaging [65].

Additive manufacturing is a promising technology that can be used to produce imaging phantoms in radiology. These phantoms can be used for training professional staff, calibrating the devices themselves, dosimetry, quality assurance, as well as evaluating and validating protocols and image processing methods. This study describes the production of an anthropomorphic phantom of a child’s head with a realistic equivalent of a CT bone count. The geometry of the created phantom resembles a paediatric patient and includes the production of bone structures with the same heterogeneous properties of bone tissue. Another strength of this study is reflected in the description of the production workflow, which includes segmentation, material selection, as well as the successful optimisation of imaging parameters in protocol for the head imaging of paediatric patients. The obtained CT number values allow the entire head phantom to be reasonably manufactured and used in clinical surroundings using a single commercially available resin and a single 3D printing device. Results and findings of the recently published articles [66,67] can spark new investigations in this area of science and can pave the way to directions of future investigations. In the first paper, the authors were able to reproduce a commercial Rando phantom with appropriate HU values for bone and soft tissue [66]. The obtained 3D-printed head phantom was suitable for use in phantom-based, patent-specific Quality Assurance (QA), which is very important in radiotherapy. In the second paper mentioned [67], the authors report on manufacturing a novel anthropomorphic head phantom for quantitative image quality assessment in cone beam computed tomography (CBCT). This is a significant step forward because the authors paid attention to the radiodensity and X-ray scattering properties of different materials used for manufacturing. These investigations will encourage similar attempts in other branches of radiology.

## 5. Conclusions

Our study demonstrated that the development of paediatric CT head imaging protocols using a 3D-printed anthropomorphic paediatric head phantom may result in a reduction in patient doses of ionising radiation without compromising diagnostic information.

By describing the SLA 3D printing technology, we created detailed anthropomorphic paediatric phantom heads using commercially available materials that simulate the radiological properties of human tissue. In conclusion, SLA printer technology can be used to produce an anthropomorphic phantom possessing complex anatomical parts that mimic the corresponding CT numbers, which in turn can be used to optimise the age-related CT protocols and significantly reduce the radiation dose delivered to the paediatric population.

Due to constant improvement in imaging techniques, there is a need for more realistic physical phantoms, both anatomical and practical. According to the results given above, our phantom lacks structural complexity, so contrast analysis studies could not be performed. Furthermore, the epoxy resin used in the manufacturing process and human brain matter do not have the same HU values, which means that the phantom could not be used in radiotherapy. The new anthropomorphic head phantom will also provide data for calculations of ionising radiation dose and its biological effects in investigations of radiation protection for patients and may therefore serve as a basis for advances in radiation safety internationally.

## Figures and Tables

**Table 1:** Scanning protocol used for adult head imaging at KCUS: tube voltage (*U*), tube current (*I*), rotation time (*t*), collimation width (*w*), pitch (*p*), slice thickness (*T*), and convolution kernel for two reconstructed series.

Mode	*U*	*I*	*t*	*w*	*p*	*T*	Conv. Kernel
Helical	120 kV	200 mA	750 ms	16 mm	0.69	3 mm/1 mm	FC26/FC30

**Table 2:** Measurements of average ($\bar{x}$), standard deviation (σ), and variance ($\sigma^{2}$) in CT numbers in the central region B of adult and manufactured paediatric phantoms at different tube voltages (*U*) and loadings (*Q*) and differences in calculated linear attenuation coefficients (Δμ/μ) between materials simulating brain tissue in two phantoms.

*U*	*Q*	$C_{\mathrm{VOL}}$ ^1^	Adult Phantom			Paediatric Phantom			Δμ/μ
(kV)	(mAs)	(mGy)	$\bar{x}$	σ	$\sigma^{2}$	$\bar{x}$	σ	$\sigma^{2}$	
80	37.0	3.60	34.7	20.3	411	48.3	10.8	118	1.31%
	75.0	7.20	33.8	14.1	199	46.2	7.74	59.9	1.20%
	112	10.8	33.6	11.5	132	46.5	6.93	48.0	1.25%
	150	14.4	33.6	9.71	94.3	46.7	5.74	32.9	1.26%
	187	18.0	33.6	8.68	75.3	48.2	5.49	30.1	1.41%
100	37.0	6.20	45.8	12.4	154	72.2	7.29	53.1	2.52%
	75.0	12.4	44.1	9.58	91.8	71.8	4.96	24.6	2.65%
	112	18.6	44.6	7.62	58.1	72.7	4.22	17.8	2.69%
	150	24.8	44.7	6.40	41.0	72.9	3.83	14.7	2.69%
	187	34.0	44.4	5.62	31.6	71.9	3.50	12.3	2.64%
120	37.0	9.10	45.7	9.97	99.4	82.0	5.68	32.3	3.47%
	75.0	18.3	46.4	7.31	53.4	81.8	3.98	15.8	3.38%
	112	27.4	47.3	6.08	37.0	81.8	3.35	11.2	3.29%
	150	36.5	45.9	4.89	23.9	82.6	2.99	8.94	3.51%
	187	50.2	45.3	4.39	19.3	81.6	2.63	6.92	3.47%
135	37.0	11.6	43.4	9.12	83.2	84.1	5.19	26.9	3.90%
	75.0	23.2	42.7	6.37	40.6	84.8	3.67	13.5	4.04%
	112	34.8	45.5	5.17	26.7	84.6	3.25	10.6	3.74%
	150	51.0	43.5	4.18	17.5	83.7	2.89	8.35	3.85%
	187	63.8	43.0	3.75	14.1	84.3	2.65	7.02	3.96%

^1^ The correlation between C_VOL_ and *σ*^2^ is significant for both phantoms (Pearson correlation test, *p* < 0.001).

**Table 3:** The look-up table for calculated values of the tube current–rotation time product (*Q*) necessary to achieve volume computed tomography air kerma index for paediatric patients ($C_{\mathrm{VOL},p}$) at different tube voltages that would produce an image with the same noise (σ or $\sigma^{2}$) in adult images at 120 kV and selected values of $C_{\mathrm{VOL}}$.

$C_{\mathrm{VOL}}$	$\sigma^{2}$	σ	$C_{\mathrm{VOL},p}$	*Q* (mAs)			
(mGy)	(HU^2^)	(HU)	(mGy)	80 kV	100 kV	120 kV	135 kV
20	52.86	7.27	7.2	74.2	46.3	33.9	28.2
30	35.50	5.96	10.4	107.9	64.0	45.8	37.3
40	27.24	5.22	13.4	139.6	80.7	57.1	46.0
50	22.42	4.74	16.3	169.7	96.4	67.8	54.1
60	19.27	4.39	19.0	198.1	111.4	77.9	61.8
70	17.04	4.13	21.6	225.1	125.5	87.5	69.1
80	15.39	3.92	24.1	250.7	139.0	96.6	76.1
90	14.11	3.76	26.4	275.1	151.8	105.3	82.7

**Table 4:** Mean values ($\bar{x}$) of scan length (*L*), tube voltage (*U*), tube current (*I*), maximum tube current ($I_{\max}$), and mean values and standard deviation (σ) of volume computed tomography air kerma index ($C_{\mathrm{VOL}}$) and air kerma length product ($P_{\mathrm{KL},\mathrm{CT}}$) in unoptimised and optimised imaging protocol for infant heads.

Protocol	Patient	Age (y)	*L* (cm)	*U* (kV)	*I* (mA)	$I_{\max}$ (mA)	$C_{\mathrm{VOL}}$^b^ (mGy)		$P_{\mathrm{KL},\mathrm{CT}}$ (mGycm)	
	n	$\bar{x}$	$\bar{x}$	$\bar{x}$	$\bar{x}$	$\bar{x}$	$\bar{x}$	σ	$\bar{x}$	σ
Unoptimised	12	0.3	159	107 ^a^	167	167	27	14	432	243
Optimised	30	0.5	171	100	170	170	21	0	365	33

^a^ The average value of four selectable options; ^b^ the difference between two protocols is significant (Student’s *t*-test, *p* = 0.021).

## Data Availability

Not applicable.

## References

[1] Goo H.W. (2012). CT radiation dose optimisation and estimation: An update for radiologists. Korean J. Radiol..

[2] Mathews J.D., Forsythe A.V., Brady Z., Butler M.W., Goergen S.K., Byrnes G.B., Giles G.G., Wallace A.B., Anderson P.R., Guiver T.A. (2013). Cancer risk in 680,000 people exposed to computed tomography scans in childhood or adolescence: Data linkage study of 11 million Australians. BMJ.

[3] Čiva L.M., Beganović A., Busuladžić M., Jusufbegović M., Awad-Dedić T., Vegar-Zubović S. (2022). Dose Descriptors and Assessment of Risk of Exposure-Induced Death in Patients Undergoing COVID-19 Related Chest Computed Tomography. Diagnostics.

[4] ICRP (2013). ICRP Publication 121: Radiological Protection in Paediatric Diagnostic and Interventional Radiology. Ann. ICRP.

[5] Winslow J.F., Hyer D.E., Fisher R.F., Tien C.J., Hintenlang D.E. (2009). Construction of anthropomorphic phantoms for use in dosimetry studies. J. Appl. Clin. Med. Phys..

[6] Okkalidis N. (2022). 3D printing methods for radiological anthropomorphic phantoms. Phys. Med. Biol..

[7] Ardila Pardo G.L., Conzelmann J., Genske U., Hamm B., Scheel M., Jahnke P. (2020). 3D printing of anatomically realistic phantoms with detection tasks to assess the diagnostic performance of CT images. Eur. Radiol..

[8] Bieniosek M.F., Lee B.J., Levin C.S. (2015). Characterization of custom 3D printed multimodality imaging phantoms. Med. Phys..

[9] Bücking T.M., Hill E.R., Robertson J.L., Maneas E., Plumb A.A., Nikitichev D.I. (2017). From medical imaging data to 3D printed anatomical models. PLoS ONE.

[10] Leng S., McGee K., Morris J., Alexander A., Kuhlmann J., Vrieze T., McCollough C.H., Matsumoto J. (2017). Anatomic modeling using 3D printing: Quality assurance and optimisation. 3D Print. Med..

[11] Mahmood U., Apte A.P., Kanan C., Bates D.D., Corrias G., Manneli L., Oh J.H., Erdi Y.E., Nguyen J., Deasy J.O. (2021). Quality control of radiomic features using 3D-printed CT phantoms. J. Med. Imaging.

[12] Meyer-Szary J., Luis M.S., Mikulski S., Patel A., Schulz F., Tretiakow D., Fercho J., Jaguszewska K., Frankiewicz M., Pawłowska E. (2022). The Role of 3D Printing in Planning Complex Medical Procedures and Training of Medical Professionals—Cross-Sectional Multispecialty Review. Int. J. Environ. Res. Public Health.

[13] Yan Q., Dong H., Su J., Han J., Song B., Wei Q., Shi Y. (2018). A review of 3D printing technology for medical applications. Engineering.

[14] Aimar A., Palermo A., Innocenti B. (2019). The role of 3D printing in medical applications: A state of the art. J. Healthc. Eng..

[15] Dukov N., Bliznakova K., Teneva T., Marinov S., Bakic P., Bosmans H., Bliznakov Z. (2020). Experimental evaluation of physical breast phantoms for 2D and 3D breast x-ray imaging techniques. Proceedings of the European Medical and Biological Engineering Conference.

[16] Ikejimba L.C., Salad J., Graff C.G., Ghammraoui B., Cheng W.C., Lo J.Y., Glick S.J. (2019). A four-alternative forced choice (4AFC) methodology for evaluating microcalcification detection in clinical full-field digital mammography (FFDM) and digital breast tomosynthesis (DBT) systems using an inkjet-printed anthropomorphic phantom. Med. Phys..

[17] Aburjaile W., Mourao A. (2017). Development of a chest phantom for testing in Computed Tomography scans. Radiat. Phys. Chem..

[18] Irnstorfer N., Unger E., Hojreh A., Homolka P. (2019). An anthropomorphic phantom representing a prematurely born neonate for digital x-ray imaging using 3D printing: Proof of concept and comparison of image quality from different systems. Sci. Rep..

[19] Leary M., Tino R., Keller C., Franich R., Yeo A., Lonski P., Kyriakou E., Kron T., Brandt M. (2020). Additive manufacture of lung equivalent anthropomorphic phantoms: A method to control hounsfield number utilising partial volume effect. ASME J. Med. Diagn..

[20] Jusufbegović M., Pandžić A., Šehić A., Jašić R., Julardžija F., Vegar-Zubović S., Beganović A. (2022). Computed tomography tissue equivalence of 3D printing materials. Radiography (Lond.).

[21] McGarry C.K., Grattan L.J., Ivory A.M., Leek F., Liney G.P., Liu Y., Miloro P., Rai R., Robinson A.P., Shih A.J. (2020). Tissue mimicking materials for imaging and therapy phantoms: A review. Phys. Med. Biol..

[22] Niebuhr N., Johnen W., Echner G., Runz A., Bach M., Stoll M., Giske K., Greilich S., Pfaffenberger A. (2019). The ADAM-pelvis phantom—An anthropomorphic, deformable and multimodal phantom for MRgRT. Phys. Med. Biol..

[23] Ali A.M., Hogg P., Johansen S., England A. (2018). Construction and validation of a low cost paediatric pelvis phantom. Eur. J. Radiol..

[24] AAPM (2015). Pediatric Routine Head CT Protocols, Version 1.1.

[25] European Commission (2014). Radiation Protection N° 180: Diagnostic Reference Levels in Thirty-Six European Countries.

[26] Awad-Dedić T., Čiva M. L., Beganović A., Busuladžić M., Đedović E., Vegar-Zubović S. (2021). Local Diagnostic Reference Levels in Emergency Computed Tomography of the Head. IFMBE.

[27] Genisa M., Shuib S., Rajion Z.A., Arief E.M., Hermana M. (2018). Density estimation based on the Hounsfield unit value of cone beam computed tomography imaging of the jawbone system. Proc. Inst. Mech. Eng. H.

[28] Lee Y.Y., Choi I., Lee S.J., Jeong I.S., Kim Y.O., Woo Y.J., Cho H.J. (2022). Clinical Significance of Gray to White Matter Ratio after Cardiopulmonary Resuscitation in Children. Children.

[29] CIRS (2013). ATOM Max Dental and Diagnostic Head Phantom Model 711-HN. https://bit.ly/3CFXdpf.

[30] Saeed M., Almalki Y. (2021). Assessment of computed tomography radiation doses for paediatric head and chest examinations using paediatric phantoms of three different ages. Radiography.

[31] Filippou V., Tsoumpas C. (2018). Recent advances on the development of phantoms using 3D printing for imaging with CT, MRI, PET, SPECT, and ultrasound. Med. Phys..

[32] Hatamikia S., Kronreif G., Unger A., Oberoi G., Jaksa L., Unger E., Koschitz S., Gulyas I., Irnstorfer N., Buschmann M. (2022). 3D printed patient-specific thorax phantom with realistic heterogenous bone radiopacity using filament printer technology. Z. Med. Phys..

[33] Ehler E., Craft D., Rong Y. (2018). 3D printing technology will eventually eliminate the need of purchasing commercial phantoms for clinical medical physics QA procedures. J. Appl. Clin. Med. Phys..

[34] Holmes R.B., Negus I.S., Wiltshire S.J., Thorne G.C., Young P., Initiative A.D.N. (2020). Creation of an anthropomorphic CT head phantom for verification of image segmentation. Med. Phys..

[35] Kikinis R., Pieper S.D., Vosburgh K.G. (2014). 3D Slicer: A platform for subject-specific image analysis, visualization, and clinical support. Intraoperative Imaging and Image-Guided Therapy.

[36] Pandzic A. (2021). Influence of layer height, build orientation and post curing on tensile mechanical properties of SLA 3D printed material. Proceedings of the 32nd DAAAM International Symposium on Intelligent Manufacturing and Automation.

[37] Chandramohan D., Cao P., Han M., An H., Sunderland J., Kinahan P., Laforest R., Hope T., Larson P. (2019). Anthropomorphic skull phantom using quantitatively accurate bone mimic material. J. Nucl. Med..

[38] Bryant J., Drage N.A., Richmond S. (2012). CT number definition. Radiat. Phys. Chem..

[39] Verdun F., Racine D., Ott J., Tapiovaara M., Toroi P., Bochud F., Veldkamp W., Schegerer A., Bouwman R., Giron I.H. (2015). Image quality in CT: From physical measurements to model observers. Phys. Med..

[40] Diwakar M., Kumar M. (2018). A review on CT image noise and its denoising. Biomed. Signal Proces. Control.

[41] Brooks R.A., Di Chiro G. (1976). Statistical limitations in x-ray reconstructive tomography. Med. Phys..

[42] Rodríguez-Sánchez Á., Thompson A., Körner L., Brierley N., Leach R. (2020). Review of the influence of noise in X-ray computed tomography measurement uncertainty. Precis. Eng..

[43] Duan X., Wang J., Leng S., Schmidt B., Allmendinger T., Grant K., Flohr T., McCollough C.H. (2013). Electronic noise in CT detectors: Impact on image noise and artifacts. AJR Am. J. Roentgenol..

[44] Ma J., Liang Z., Fan Y., Liu Y., Huang J., Chen W., Lu H. (2012). Variance analysis of x-ray CT sinograms in the presence of electronic noise background. Med. Phys..

[45] Karappara J., Koteshwar P., Panakkal N.C., Sukumar S. (2020). Optimization of paediatric CT brain protocol to achieve reduced patient dose. Biomed. Pharmacol. J..

[46] Jaramillo-Garzón W., Caballero M., Alvarez-Aldana D. (2021). Size-specific dose estimates for paediatric non-contrast head CT scans: A retrospective patient study in Tunja, Colombia. Radiat. Prot. Dosim..

[47] Carmichael J., Moores B., Maccia C. (2000). European Guidelines on Quality Criteria for Diagnostic Radiographic Images.

[48] Vasung L., Turk E.A., Ferradal S.L., Sutin J., Stout J.N., Ahtam B., Lin P.Y., Grant P.E. (2019). Exploring early human brain development with structural and physiological neuroimaging. Neuroimage.

[49] Brooks T., Choi J.E., Garnich M., Hammer N., Waddell J.N., Duncan W., Jermy M. (2018). Finite element models and material data for analysis of infant head impacts. Heliyon.

[50] Bartholomeusz H., Courchesne E., Karns C. (2002). Relationship between head circumference and brain volume in healthy normal toddlers, children, and adults. Neuropaediatrics.

[51] Pearce M.S., Salotti J.A., Little M.P., McHugh K., Lee C., Kim K.P., Howe N.L., Ronckers C.M., Rajaraman P., Craft A.W.S. (2012). Radiation exposure from CT scans in childhood and subsequent risk of leukaemia and brain tumours: A retrospective cohort study. Lancet.

[52] Gao Y., Quinn B., Pandit-Taskar N., Behr G., Mahmood U., Long D., Xu G.X., Germain J.S., Dauer L.T. (2018). Patient-specific organ and effective dose estimates in paediatric oncology computed tomography. Phys. Med..

[53] Spampinato M.V., Stalcup S., Matheus M.G., Byington K., Tyler M., Bickley S., Tipnis S. (2018). Radiation dose and image quality in paediatric head CT. Radiat. Prot. Dosim..

[54] Mohammadbeigi A., Khoshgard K., Haghparast A., Eivazi M.T. (2019). Local DRLs for paediatric CT examinations based on size-specific dose estimates in Kermanshah, Iran. Radiat. Prot. Dosim..

[55] Abdulkadir M.K., Shuaib I.L., Achuthan A., Nasirudin R.A., Samsudin A.H.Z., Osman N.D. (2022). Estimation of paediatric dose descriptors adapted to individual specific size from CT examinations. Radiat. Prot. Dosim..

[56] Granata C., Origgi D., Palorini F., Matranga D., Salerno S. (2015). Radiation dose from multidetector CT studies in children: Results from the first Italian nationwide survey. Pediatr. Radiol..

[57] Célier D., Roch P., Etard C., Le Pointe H.D., Brisse H.J. (2020). Multicentre survey on patient dose in paediatric imaging and proposal for updated diagnostic reference levels for France. Part 1: Computed tomography. Eur. Radiol..

[58] EuropeanCommission (2018). European Guidelines on Diagnostic Reference Levels for Paediatric Imaging.

[59] Burton C.S., Szczykutowicz T.P. (2018). Evaluation of AAPM Reports 204 and 220: Estimation of effective diameter, water-equivalent diameter, and ellipticity ratios for chest, abdomen, pelvis, and head CT scans. J. Appl. Clin. Med. Phys..

[60] Keat N. (2005). CT scanner automatic exposure control systems. MHRA Rep. 05016.

[61] Söderberg M., Gunnarsson M. (2010). Automatic exposure control in computed tomography–an evaluation of systems from different manufacturers. Acta Radiol..

[62] Tack D., Kalra M.K., Gevenois P.A. (2012). Radiation Dose from Multidetector CT.

[63] Inoue Y., Itoh H., Shiibashi N., Sasa R., Mitsui K. (2022). Sample Size and Estimation of Standard Radiation Doses for Pediatric Brain CT. Tomography.

[64] McNitt-Gray M.F. (2002). AAPM/RSNA physics tutorial for residents: Topics in CT: Radiation dose in CT. Radiographics.

[65] Abdulkadir M.K., Rahim N.A.Y.M., Mazlan N.S., Daud N.M., Shuaib I.L., Osman N.D. (2020). Dose optimisation in paediatric CT examination: Assessment on current scanning protocols associated with radiation dose. Radiat. Phys. Chem..

[66] Kim S.Y., Park J.W., Park J., Yea J.W., Oh S.A. (2022). Fabrication of 3D printed head phantom using plaster mixed with polylactic acid powder for patient-specific QA in intensity-modulated radiotherapy. Sci. Rep..

[67] Wang Y., Dankelman J., Ruijters D. (2022). Cost-efficient anthropomorphic head phantom for quantitative image quality assessment in cone beam CT. Biomed. Phys. Eng. Express.

